# Road traffic injuries in one local health unit in the Lazio region: results of a surveillance system integrating police and health data

**DOI:** 10.1186/1476-072X-8-21

**Published:** 2009-04-22

**Authors:** Francesco Chini, Sara Farchi, Ivana Ciaramella, Tranquillo Antoniozzi, Paolo Giorgi Rossi, Laura Camilloni, Massimo Valenti, Piero Borgia

**Affiliations:** 1 Technology assessment unit, Agency for Public Health, Rome, Italy; 2 Prevention department, Local Health Unit RMB, Rome, Italy; 3 Faculty of Medicine "La Sapienza" University, Rome, Italy

## Abstract

**Objective:**

Different sources are available for the surveillance of Road Traffic injuries (RTI), but studied individually they present several limits. In this paper we present the results of a surveillance integrating healthcare data with the data gathered by the municipal police in the southeastern area of Rome (630,000 inhabitants) during the year 2003.

**Methods:**

The Municipal police RTI reports, which list the exact location, circumstances and some risk factor of the crash, were searched in the emergency visit, hospitalization and mortality databases, to integrate them with the information on health consequences.

A multivariate analysis was conducted to evaluate risk factors (crash circumstances, age ad gender of the casualty) associated with hospital admission following a RTI.

Mapping of RTI locations was created. The locations with higher risk of accidents with severe health consequences and at higher risk for pedestrians were identified.

**Results:**

According to police records 4571 RTI occurred in 2003, 75% of which led to emergency department admissions. Sixteen percent of these emergency visits ended in hospitalization, and 44 deaths were reported within 30 days of the event, most of which occurred in young men.

The people with the highest risk of hospitalization after an RTI were the cyclists, pedestrians and followed by people on two-wheeled vehicles. The type of crash with the highest risk of hospitalization was head-on collision.

Geographical analyses showed four clusters with higher severity of RTI. Specific attention was paid to pedestrian injuries. Analyzing the locations of RTIs involving pedestrians permitted us to rank the most dangerous streets. The roads at high risk for pedestrians identified problems in the bus stop constructions and in the placement of the zebra pedestrian crossings.

**Conclusion:**

This study proves the feasibility of an integrated surveillance system of RTI by using routinely collected local data. The high-risk locations identified with the geographic analyses method in this study highlighted infrastructural problems, suggesting immediate preventive interventions.

## Introduction

Road traffic injuries (RTI) are the greatest cause of death in young adults in industrialized countries [1].

Preventing traffic injuries in industrialized and developing countries is one of the primary challenges of the World Health Organization [2]. In fact, the European Union has set the objective of reducing traffic accident deaths 40% reduction in traffic accident deaths by 2010 [3].

Accurate information about this phenomenon and its health consequences is fundamental for monitoring the effectiveness and to readdress policies. In particular it is important that the institute or agency that collects the information is in direct contact with policy makers and serves as a public advocate.

In Italy the data on the number of injuries, deaths, and accidents are recorded in police reports, which since they are designed for legal instead of medical purposes, report very little information on the health consequences of the people involved in RTIs.

Health information can be obtained from existing information systems (hospital discharge reports and mortality). In addition, some regions have recently improved their information systems by adding data from emergency department visits.

These different sources available for the surveillance of RTI, taken individually, present limits: due to the lack of data about the event reported in the health information, it is not possible to geo-reference the location of the accident, whereas the police data does not include any medical information.

Therefore, integrating these sources is indispensable to build an accurate surveillance system that can track the circumstances of a traffic accident, the risk factors and the medical consequences, as well as establishing and evaluating prevention interventions [4]

An RTI surveillance system has been active in the Lazio region since the year 2000, which integrates data from emergency departments with hospital discharge and mortality records [5]. The local health unit (LHU) RM/B in the city of Rome (2,700,000 inhabitants) for the years 2002–2004 began collaborating with the municipal police to collect and examine traffic accident reports that occurred in their urban area, in order to plan prevention activities.

The goal of this work is to test the feasibility and to validate a road traffic injury surveillance system integrating healthcare surveillance already in operation, with the data gathered by the municipal police for the southeastern part of Rome.

## Methods

### Setting

The pilot surveillance system was implemented in the southeastern zone of Rome in a territory covered by one Local Health Unit (RM/B LHU). The LHUs in Italy are geographically-based organizations responsible for assessing the medical needs and providing comprehensive care for a defined population. The LHU is responsible for the hospital and primary care services in its area and for prevention and health promotion activities. The RM/B LHU has 630,000 inhabitants (24.3% of the total population of Rome), and covers a section that runs from the city center to poorer outskirts of the city.

### Data sources

#### Health care information

The Emergency Information System (EIS): collects information on all visits to emergency departments in the Lazio region since 1999. The information collected includes the patients' demographics, the mode of arrival, treatment urgency (triage code), procedures, diagnoses, and outcome (discharged home, transferred, admitted, died). In case of trauma, the type or location of the accident is also reported (intentional violence, road, work, home, other). The triage code is an urgency scale commonly used in crowded emergency departments to determine which patients should be seen and treated immediately. Triage code involves a color-coding scheme using red-urgent, yellow-critical, green- deferrable, white-not appropriate for emergency department.

The Hospital Discharge (HD): has gathered and managed data from all hospital admissions each year since 1994 from both public and private hospitals in the region.

Mortality Registry (MR): collects information about deaths that occur in the Lazio region and on all the deaths of residents, regardless of where the deaths occur. The registry records: name, last name, date and place of birth, place of residence, date and place of death, and cause of death (ICD-9 code).

These three data sources have been integrated in a unique surveillance system; the methods used for this surveillance are described elsewhere [5]. Briefly, each road trauma patient listed by in the EIS archives was searched in the HD for subsequent hospitalizations using name, birthday and date of admission (+/- 24 h) as record linkage keys.

The EIS-HD integrated database was linked to the MR to identify deaths that occurred within 30 days of the first emergency visit. The cases present in the mortality registry that reported external causes of traffic accident death (E800-819; E826; E829) that were not listed in the EIS or HD were considered as deaths that occurred at the accident location.

#### Municipal police RTI report form

Police RTI reports were recorded using a standardized form (verbatel, inc) used by several police forces in Italy. These reports record information on all accidents that occur in the study area, involving residents and non-residents, and list the date, time, circumstances, and location of the accident, environmental factors, types of vehicle, age, gender and position of the passengers. The health information reported is not specific, and indicates only injury or death. The site of each RTI was obtained by using the longitude and latitude coordinates of each address using a ARCGIS map function.

Data were available for the year 2003.

#### Linkage between health care surveillance and police RTI reports

The integrated healthcare database, obtained following the above-reported procedures, was linked to the database containing information collected by the municipal police, by implementing a stepwise strategy for linkage using demographic linkage keys. The linkage was performed using the SAS statistical package.

### Analyses

The new integrated database listed the number of RTI with injuries, emergency department visits, admissions and deaths, as well as the type of injury reported and the body region affected; these last two were identified by reclassifying the trauma diagnoses using the Barell matrix [6].

The incidence of RTI-related emergency visits was estimated using all visits involving RM/B LHU residents that occurred in any of the EDs in Lazio as the numerator, and the resident population in the same area as the denominator.

Mapping of RTI locations was created using ARCGIS software [7]. This analysis allows us to view, understand, question, interpret, and visualize data in many ways that reveal relationships, patterns, and trends in the form of maps, globes, reports, and charts.

Specifically, we created five maps: the first map described RTIs according to injury severity (triage code); the second plotted pedestrian RTIs according to the vehicle struck; the third mapped neighborhoods in order of number of pedestrian RTIs per 100.000 inhabitants; the fourth map reports the rank of neighborhoods by RTIs according to road length; the last map classified the road arcs according to the number of RTIs involving pedestrians.

The risk factors associated with hospital admission (as a proxy of the severest cases) following a traffic injury were evaluated using logistic regression models.

The logistic model was constructed adjusting for gender and age and tested three exposure variables together: crash type (head on collision, frontal-lateral collision, lateral collision, "fender bender" of moving vehicle, pedestrian run over by vehicle), position of injured person (car-driver, car-passenger, public transport, moped-driver, moped-passenger, motorbike-driver, motorbike-passenger, lorry, pedestrian, bicycle), vehicle/object struck (car, public transport, moped, motorbike object)

The choice of the variable was made a priori. The models were constructed using the STATA statistical package.

## Results

### The dataset

The integrated health surveillance system recorded 157,089 emergency department visits and 11,799 (7.5%) of these patients were hospitalized from RTIs in the Lazio region, while 701 died within 30 days of the accident.

There were 23.214 emergency visits involving residents of the study area (i.e. the south east sector of Rome, the RM/B LHU), with the highest incidence of emergency department visits for RTI in the Lazio region (3685/100.000 inhabitants CI 95% 3638–3731; Regional incidence 2847/100.000 inhabitants CI 95% 2833–2862), there were 1349 hospitalizations (incidence 213/100.000 inhabitants), and 153 deaths (mortality 24/100.000 inhabitants).

The municipal police database reported 3,497 accidents involving 4,571 injured persons. About two- thirds (65%) of the injured were male, the average age was 34, and the median 31 years old.

**Figure 1:**
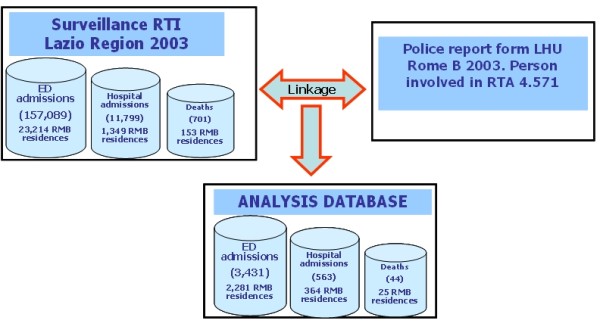
**Integration of health and police data**. LHU Rome B year 2003.

As a result of the integration with the healthcare surveillance system (figure 1) it was possible to identify emergency department visits for 75% (3431) of the cases reported by the police, resulting from 2,878 traffic accidents. The incidence rate based on police reports is almost 5 times lower than the incidence rate reported by emergency departments.

Eighty percent (2768) of linked cases were obtained in the first step of linkage without clerical work, which utilized all demographic variables available as the linkage keys, and considered up to one day delay between date of accident and date of emergency department visit. After a clerical review of a 10% sample of the linked records, the true match rate was 100%, as it is a deterministic linkage procedure.

### Determinants of severity

Sixteen point four percent of emergency department visits ended in hospitalization, and 44 deaths within 30 days of the accident were reported, most of which occurred in young men. The parts of the body most frequently involved were the spine and back (23.3%) and legs (18.1%). More than a third of the injuries were contusions, and a considerable portion of injuries were fractures (11.2%). Almost five percent (4.8%) of emergency department visits did not report a diagnosis of trauma (table 1).

**Table 1:** Injuries by body region and nature of the injury for patients with emergency department admission – LHU Rome B year 2003

**BODY REGION**	**N**	**%**
Spine and back	798	23.3
Legs	621	18.1
Arms	503	14.7
Multiple	429	12.5
Head & neck	366	10.7
Brain injuries	239	7.0
Torso	177	5.2
No trauma diagnosis*	166	4.8
Undefined	123	3.6
Systemic	9	0.3

**Total**	**3431**	**100.0**
		
**NATURE OF THE INJURY**	**N**	**%**

Contusion/superficial	1207	35.2
Sprains and strains	631	18.4
Fracture	385	11.2
Multiple	334	9.7
Undefined	256	7.5
Internal injury	244	7.1
No trauma diagnosis*	166	4.8
Open wounds	125	3.6
Crushes/amputation	35	1.0
Dislocation	31	0.9
Systemic	9	0.3
Burns	7	0.2
Blood vessel injury	1	0.0

**Total**	**3431**	**100.0**

* ICD-9-CM code 001-799

Drivers, regardless of vehicle type, accounted for the vast majority of RTI emergency department visits.

Pedestrian, particularly when struck by a lorry, reported a higher percentage of red and yellow triage and deaths. Cyclist and pedestrian when struck by a car reported the higher percentages of of hospital admissions. Car-passengers were less seriously injured, regardless of category (table 2).

**Table 2:** Position of injured persons and health outcomes by type of vehicle involved in the accidents – LHU Rome B year 2003

**Car**								
**POSITION**	**Emergency department admissions (EDA)**		**Red and yellow triage code**		**Hospital admissions**		**Deaths**	

	N	%	N	% on EDA	N	% on EDA	N	% on EDA
	
Driver	1198	68.3	110	9.2	118	9.8	8	0.7
Passenger	337	19.2	20	5.9	29	8.6	2	0.6
Pedestrian	220	12.5	74	33.6	82	37.3	12	5.5

**TOTAL**	**1755**	**100.0**	**204**	**11.6**	**229**	**13.0**	**22**	**1.3**

**Lorry**								

**POSITION**	**Emergency department admissions (EDA)**		**Red and yellow triage code**		**Hospital admissions**		**Deaths**	

	N	%	N	% on EDA	N	% on EDA	N	% on EDA
	
Driver	100	71.9	29	29.0	21	21.0	2	2.0
Passenger	27	19.4	5	18.5	4	14.8	1	3.7
Pedestrian	12	8.6	5	41.7	4	33.3	1	8.3

**TOTAL**	**139**	**100.0**	**39**	**28.1**	**29**	**20.9**	**4**	**2.9**

**Two-wheelers including bicycle**								

**POSITION**	**Emergency department admissions (EDA)**		**Red and yellow triage code**		**Hospital admissions**		**Deaths**	

	N	%	N	% on EDA	N	% on EDA	N	% on EDA
	
Driver	1279	86.9	297	23.2	245	19.2	13	1.0
Passenger	78	5.3	13	16.7	10	12.8	1	1.3
Pedestrian	87	5.9	24	27.6	24	27.6	3	3.4
Cyclist	28	1.9	10	35.7	10	35.7	1	3.6

**TOTAL**	**1472**	**100.0**	**344**	**23.4**	**289**	**19.6**	**18**	**1.2**

The most common types of RTI were collisions and fender benders of moving vehicles, which together accounted for 78% of the occurrences.

**Figure 2:**
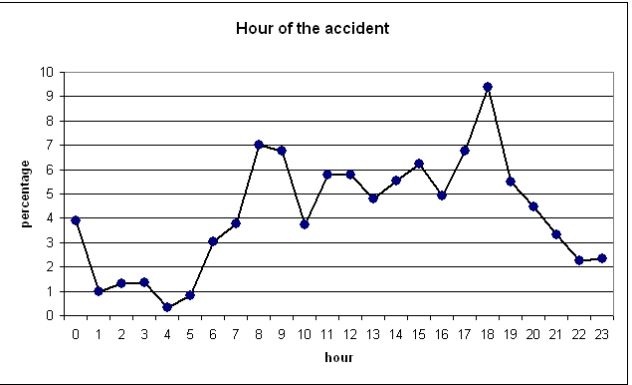
**Road Traffic Injuries by the hour of the accident**. LHU Rome B year 2003.

The weather conditions were clear and dry in 79% of traffic accidents, which is confirmed by dry road conditions at 88% of the crashes. Most injuries occurred during rush hour traffic, between 9 and 10 am, and at 7 pm (figure 2).

The multivariate analysis (table 3) showed that patients injured in a front/lateral, fender-bender and lateral accident presented a lower risk of hospitalization than those injured in head-on collisions.

**Table 3:** Risk factors associated with hospitalisation.

	**Number hospital admissions**	**OR**	**95% CI**
**CRASH TYPE**			
head on collision	54	**1**	
frontal-lateral collision	193	0.54	0.37–0.78
lateral collision	56	0.37	0.24–0.58
fender bender of moving vehicles	53	0.36	0.23–0.56
run over by vehicle (pedestrian)	110	1.47	0.49–4.41
**POSITION**			
car driver	118	**1**	
car passenger	29	1.09	0.69–1.71
public transport	1	1.39	0.16–11.76
moped driver	97	2.18	1.60–2.97
moped passenger	9	2.26	1.01–5.07
motorbike driver	148	2.14	1.61–2.85
motorbike passenger	1	0.64	0.08–4.89
lorry	25	1.93	1.16–3.21
pedestrian	110	2.30	1.12–6.48
bicycle	10	5.02	2.13–11.84
**OPPONENT VEHICLE STRUCK**			
car	383	**1**	
public transport	7	1.22	0.50–2.98
moped	22	0.98	0.57–1.69
motorbike	18	0.56	0.32–0.97
lorry	43	1.64	1.12–2.39
vehicle against obstacle	72	2.12	1.57–2.86
**SEX**			
male	436	**1**	
female	127	0.52	0.41–0.66
**AGE CLASS**			
0–4	3	**1**	
5–14	19	1.28	0.31–5.27
15–34	301	1.31	0.35–4.93
35–64	187	1.44	0.38–5.44
>= 65	53	1.58	0.41–6.10

ORs and 95% confidence intervals estimated using logistic regression models – LHU Rome B year 2003.

Cyclists and pedestrians reported the highest risk of being hospitalized, OR 5.02 95%CI 2.13–11.84 and OR 2.30 95%CI 1.12–6.48, respectively; lorry users showed a higher risk of hospitalization compared to motorists.

To be struck by a lorry or to hit an obstacle was associated with a higher risk of hospitalization than to be struck by an automobile.

### Geographical analysis

**Figure 3:**
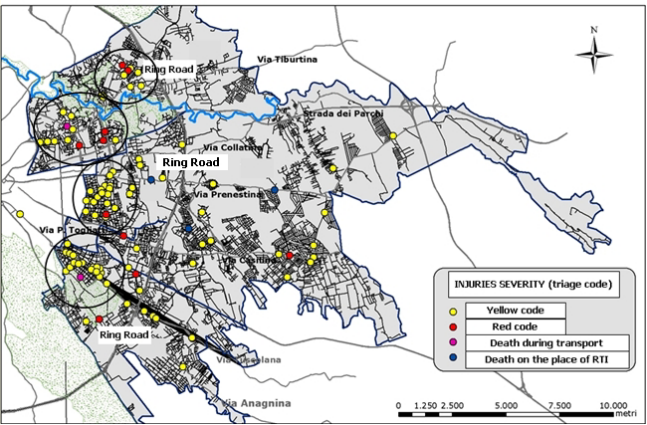
**Geographic Distribution of RTI for severity (triage code)**. LHU Rome B year 2003.

The map reported in figure 3 shows the plot of the most severe RTIs in the study area. Surprisingly most of the severe RTIs were located in the left part of the map, corresponding to the area inside the Roman ring road (Grande Raccordo Anulare), in the center of the city. In particular, four clusters were identified, all in the inner area.

**Figure 4:**
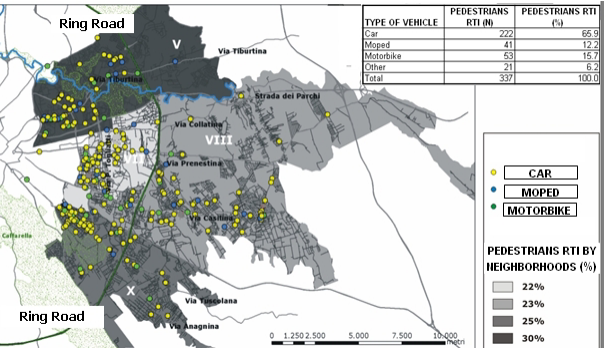
**RTIs involving pedestrian**. LHU Rome B year 2003. In the map are plotted the pedestrian RTI by type of vehicle struck.

**Figure 5:**
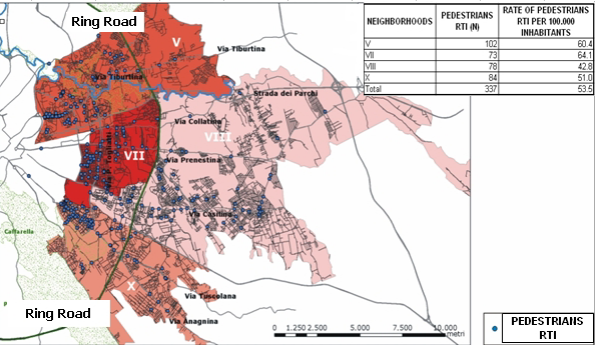
**RTIs involving pedestrian**. LHU Rome B year 2003. In the map the neighbourhoods are classified according to the rank of pedestrian RTI per 100.000 inhabitants

**Figure 6:**
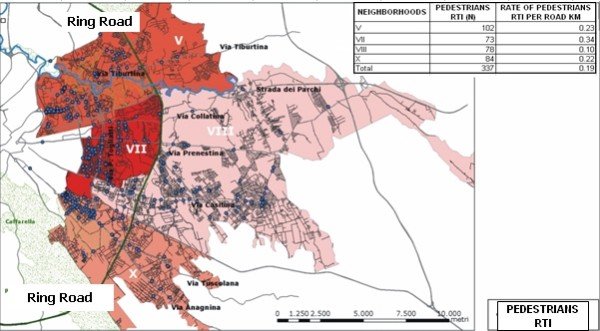
**RTIs involving pedestrian**. LHU Rome B year 2003. In the map the neighbourhoods are classified according to the rank of pedestrian RTI per road length

Specific attention was paid to pedestrian injuries, as suggested by the analyses of the risk factors, but also as suggested by the policy makers themselves. In figure 4, darker areas represent higher numbers or higher rates of accidents (figure 5, figure 6). We plotted the injuries according to the place of the accident (figure 4) most of the accidents were caused by cars, followed by motorbikes and mopeds. The neighborhood V reported the most RTIs involving pedestrians, but the highest number of accidents where pedestrians were struck by a car occurred in neighborhood X.

**Figure 7:**
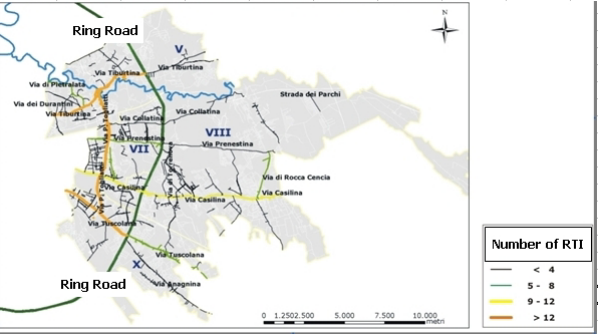
**RTIs involving pedestrian**. LHU Rome B year 2003. In the map the road arcs are classified according to the pedestrian RTI.

**Figure 8:**
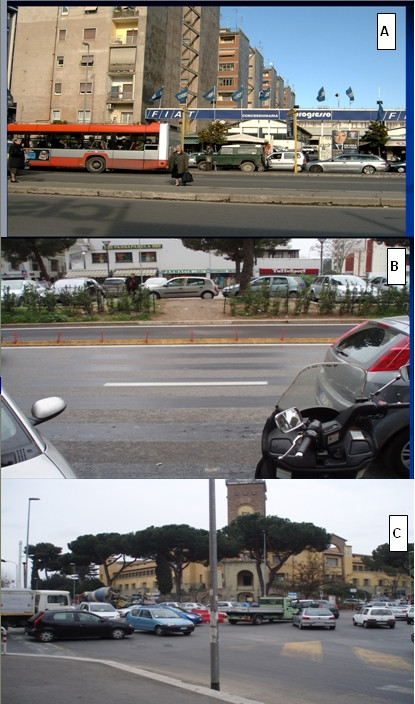
**RTIs involving pedestrians – high risk roads**. LHU Rome B year 2003. The picture shows a bus stop without any zebra crossing connecting the platforms and that the zebra crossing was removed, but the signs on the road pavement are still evident The last picture (C)shows crowded crossing with poor traffic signs for both vehicles ad pedestrians.

Figures 5, figure 6 show that the rank of neighborhoods according to the number of injured pedestrians per 100.000 inhabitants and according to the number of injured pedestrians road length is the same (VII, V, X, VIII), but is substantially different from the rank according to the absolute number of accidents involving pedestrians (V, X, VIII, VII). Finally, figure 7 classifies the streets according to the number of injured pedestrians: 3 roads had more than 12 accidents involving pedestrians, 2 roads had from 9 to 12, seven roads from 5 to 8, 36 roads from 2 to 4, 103 roads had only one. Analyzing the locations of RTIs involving pedestrians permitted us to rank the most dangerous streets. This ranking was compared with the list of the most dangerous roads published by the municipality. The same three roads were identified by both sources to be the most dangerous in the study area, but received different rankings according to the different sources: Via Tiburtina (1st and 3rd), Viale Palmiro Togliatti (2nd and 1st) and Via Tuscolana (3rd and 2nd). Specific site visits found that these streets lack important traffic indicator signs. Figure 8 shows three examples of roads at high risk for RTI involving pedestrians due to poor traffic signage. In both cases, the hazards of the locations are apparent and suggest the appropriate preventive intervention.

## Discussion

This study demonstrates the feasibility of an integrated RTI surveillance system using commonly available data, from healthcare organizations and police reports, with the aim of directing prevention actions.

Similar integrated RTI surveillance systems have been implemented both in Italian [8,9] and international contexts. Particularly significant are the western Australia studies [10,11]. However, in some contexts the tests gave negative results, mostly due to a lack of demographic variables in the available data.

This study showed that head on collisions are the most dangerous, while the groups most in danger are the most vulnerable on the road: pedestrians, motorcyclists and cyclists. [12]. The use of security devices like seat belts and airbags, not to mention the better structural features of automobiles, notably increased the safety of drivers and passengers. On the contrary, less attention has been given to the safety of those most vulnerable subjects on the road, for whom solutions could be developed that limit the damage caused from automobile impact, in addition to improving urban planning [13].

The higher risk of hospitalization emerged for the bicycle users could be due to an under reporting of minor cyclists accidents by the police, in particular when the third party involved in the accidents is less [14].

Surprisingly the vast majority of the severe accidents occurred within the ring road, i.e. in a densely urbanized area, where speed is supposed to be low. This may be due to the high prevalence of weak road users in Rome (particularly moped and motorbike users [15]), compared to other cities in industrialized countries. Accidents involving these vehicles, as well those involving pedestrians, may have severe consequences even when the force of the impact is not strong. The neighborhood ranking of RTI changes when we use absolute numbers, RTIs per 100.000 inhabitants or RTIs by road length. Neighborhood VIII in particular, which is a less urbanized area, had a 20% lower rate of pedestrian accidents per 100.000 inhabitants and 50% fewer pedestrian injuries per Km of road than the mean of the study area. The most dangerous streets identified by our surveillance are the same listed as the most dangerous by the municipality's Urban Traffic Plan document [16], but with a different rank.

According to the results of the geographical analysis, LHU officers made site visits to the roads where clusters of pedestrian RTIs occurred. The results of these site visits were very useful in providing evidence of a causal relationship between structural deficiencies, in particular of lack of traffic signage, and accidents. This process of surveillance, analysis, comprehension of the causal relationship and communication with policy makers, is, in our opinion a good example of the advocacy function of the Health Service, who together with the police, can improve road safety.

The development of this integrated system with geographical analysis added an important qualitative element [17], making it possible to evaluate the danger level of roads and streets in the area, not only by observing the number of injuries that occurred on a specific street or area, but also by considering the severity of the consequences, i.e. to distinguish between heavily-trafficked areas where crashes lead only to superficial lesions, from others where injuries are usually more severe.

The element necessary to create a map of risk that considers health outcomes is the availability of nominative data to correctly match the health information with the data collected by the local police. If the nominative data are not available for privacy reasons, techniques of probability-based record linkage can be implemented [18,19], yet even those require the availability of other information on individuals, like gender and age of the accident victim, and those relative to the accident, such as the date of the accident and emergency department visit.

Nevertheless, the geographical analysis provides useful information even without an integrated surveillance, because one of the two maps we present, the one that cites the location of accidents involving pedestrians, can be constructed using only police reports.

### Limits

Incidence definitions: resident cases on resident people or cases occurred in the area on resident population. Most of the routine statistics use the second definition even though can be inaccurate because the numerator may include some cases not present in the denominator. Unfortunately, the statistics based on the health information system do not precisely list the location of the crash making it impossible to consider location in the incidence definition. We used the first definition for incidence, but the police reports (for which we do not have a denominator) were selected based on the location of the crash.

The health care surveillance system definition is "injuries that occurred on the road"; it may also include injuries not caused by any vehicle, while it does not include work injuries or intentional injuries.

This discrepancy could overestimate incidence in very old people, for whom injuries from falls are common [20]. However, this problem is not present in the database that was integrated with police reports, because we only considered accidents involving at least one vehicle.

Some injuries may have gone undetected by both systems. Underreporting in police reports is well known and is relevant for less severe cases, but also has occurred with deaths and very severe cases. On the other hand, underreporting by emergency systems is more difficult to measure and probably is relevant for the less severe injuries (those not requiring hospital treatment, even though GPs in Italy are not usually consulted for traumas), and for work-related RTIs that can be reported as "work" injuries and not "road" injuries. In our opinion, the combination of the two sources, i.e. the police dataset and the emergency dataset, is a very sensitive surveillance system, and while its specificity remains unclear, it is certainly the most sensitive we have at the present time.

In this paper we used both red/yellow triage and hospitalization as outcomes, a choice that suggests urgent triage and hospitalization are proxies of injury severity. We know that there are cases in which urgency is poorly related to severity, but on average there is a very strong correlation between triage code and injury severity score [21]. Also, hospitalization after an emergency visit is influenced by many factors, some of which have nothing to do with the severity of the injury. Nevertheless this can be considered a difficult outcome from a health service point of view.

The models do not represent the risk of hospitalization for an RTI for any exposure, but the risk of hospitalization given an injured subject, consequently the exposure must be interpreted as a determinant of the severity and not of the risk of injury.

Another important limit of our study concerns the absence of information on the number of alcohol and drug-related RTIs [22]. This information was incomplete in the health database, and was not disclosed to us in the police reports. This limit however does not influence the geographical analysis.

Finally, our study did not consider some important confounders of ED visits such as distance, socio-economic factors and so on [23,24]. However, road traffic injuries do not tend to be affected by these factors, especially in our country where immediate care is offered by the ED free of charge.

## Conclusion

This study indicates the feasibility of an integrated system for RTI. Identifying locations of increased risk for severe health consequences suggested targeted preventive interventions that are very likely to be effective.

This project involved prevention departments of the RM/B LHU and the police department, which began to collaborate in order to initiate evidence-based prevention activities, such as rotaries, traffic calming zones, speed bumps, and intensifying traffic controls.

## Competing interests

The authors declare that they have no competing interests.

## Authors' contributions

FC, SF, PGR and PB conceived and designed the study. FC, SF and IC planned the strategy of analysis. FC and LC performed the statistical and epidemiologic analysis. AT, IC and MV performed geographical analysis. FC, SF and PGR drafted the paper. All authors read and approved the final manuscript.
